# Individual investment decision behaviors based on demographic characteristics: Case from China

**DOI:** 10.1371/journal.pone.0201916

**Published:** 2018-08-09

**Authors:** Qiujun Lan, Qingyue Xiong, Linjie He, Chaoqun Ma

**Affiliations:** 1 Business School, Hunan University, Changsha, Hunan, China; 2 Business School, Hunan Normal University, Changsha, Hunan, China; Central South University, CHINA

## Abstract

Predicting and analyzing behaviors of investors is of great value to financial institutions. This paper uses survey data from about 9,000 individual investors across China to explore the predictability of decision behaviors by studying demographic characteristics that are relatively easy to obtain. After applying Pearson’s chi-squared test, Spearman rank correlation test, and several data mining methods, we verified that demographic characteristics are closely linked to decision behaviors, and it would be an economical and feasible solution for financial organizations to build initial behavioral prediction models especially when investors’ behavioral data are insufficient.

## Introduction

In the early 1990s, China’s stock exchanges (one in Shanghai and another in Shenzhen) were officially established as an experiment for market economy reform. After more than 20 years of rapid development, the China’s financial market system is becoming increasingly more mature. In the meantime, the vitality of financial investors has been enhanced greatly [1]. Financial institutions local and abroad are aware that China will be a vast market and competition will be vigorous. Facing these challenges, many companies are eager to get insights into the Chinese financial market and investors. Much different from most developed financial markets where institutional investors are majority, individuals account for nearly 80% of all investors in China [2]. And by 2017, data from the China Clearance Center showed that the number of individual investors in the A-share stock market amount to over 133 million and one out of every ten people in China invests in the stock market.

As we know, precision marketing and personalized service have been hot topics and key strategies for firms to gain a competitive advantage in the era of Big Data. In response to such a huge and important group of investors, an interesting question is, “what kind of investment behavior preferences do individual investors have in China’s financial market?” The answers to these questions can directly influence the strategies of service providers. For example, based on clients’ preference and tolerance of risk, a brokerage firm can target them with a risky product. Accordingly, gaining insights into investors’ behavior preferences becomes a necessary measure for companies to develop new customers, reduce management costs, provide personalized services, and to obtain a competitive advantage [3].

However, investors’ behavior preferences are hard to observe and measure directly due to their dynamics, ambiguity, heterogeneity and uncertainty [4]. It is difficult even for institutions to obtain such information from individual investors due to legal restrictions. However, some personal characteristics of investors such as demographic characteristics are much easier to access and measure legally than the behavioral information. Applying some easy-to-obtain personal characteristics as predictors to build models to evaluate investors’ behavior preferences may be a solution to this problem.

This paper aims to analyze the capability to predict investment behaviors based on some demographic characteristics, and verify the feasibility and effectiveness of building behavior prediction models based on these characteristics. To fulfill these purposes, we collected survey data from more than 20,000 Chinese individual investors and used several methods to analyze predictability. The paper proceeds as follows: firstly, it reviews the literature relevant to financial behavior preferences and personal characteristics; secondly, the variables and data are described; thirdly, we provide the methodology used to analyze predictive capability and build a prediction model, then present the results; to illustrate the validity of the model, an assumed application case is provided in the fourth section; finally, the conclusions are drawn.

## Literature reviews

Traditional finance theories such as the “Efficient Market Theory” and “Modern Portfolio Theory” hold that the investors’ behaviors are rational and logical, and all activities are reflections of economic information [5]. Scholars like Kahneman and Tversky [6] established and developed behavioral finance theories. According to the view of Pompian [7], these theories can be divided into two types, Behavioral Finance Macro and Behavioral Finance Micro. The former usually studies institutional investors, and the latter mainly concerns the individual investors. The original intention of focusing on Behavioral Finance theories was to explain stock market anomalies and market bubbles and crashes in order to increase the efficiency of financial markets by applying psychology and other social science theories [8]. In the course of this research, scholars observed personal characteristics and behaviors from the perspective of an investment effect. Extensive personal characteristics of investors were involved, such as personality, genetic characteristics, education, social position, economic capability, experience, emotion, cognition, etc. For example, Pompian and Longo [9] investigated 100 investors using the Myers-Briggs personality test list and questionnaire, and found that there were striking differences among individual investors with different preferences including preferences of investment types, choices of information channels and trading behaviors. Wen [10] built a D-GARCH-M model to examine the relation between Investors’ Risk Preference and return on stock market, found that investors become risk aversion when they gain and risk seeking when they lose and the extent of risk aversion in gains and that of risk-seeking in losses were different. Clark-Murphy and Soutar [11] applied cluster analysis and discriminant analysis in their study and divided the samples into four categories according to the different attitudes and decision-making behaviors of individual investors, and found that individual investors in each category have different features in investment preferences and target selections. Hira and Loibl [12] paid special attention to differences of investment behavior caused by gender, and they found that gender had an impact on the acquisition sources of investment information and risk-taking level by conducting a national randomized sample and telephone interview. Barnea et al [13] used twin investors’ investment records (which were very difficult to obtain) to discuss the linkages among individual investor’s characteristics, market participation habits and capital investment distribution behaviors with Pearson’s correlation test. The authors found that one third of investment behavior differences can be explained by individual genetic characteristics. Kabra et al [14] found people of different ages and genders have varying risk tolerance levels in decision making processes by factor analysis and regression analysis. Cary Frydman et al [15] conducted a study using functional magnetic resonance imaging to test a “realization utility” explanation for their behaviors.

As this paper is related to the Chinese financial market, some related literature about the market are as follows. At an early stage of the stock market, Xinghui and Xiaohong [16] made an investigation in Shanghai and found that all of personal characteristics, abilities, social and economic environments could influence stock investment performance. Bojin [17] made a questionnaire survey on the individual investors and institutional investors of 126 sales departments in Jiangsu Province, aimed at understanding the factors that affect their investment behaviors, including their composition situations, psychological qualities, investment techniques, as well as politics, economies, policies, information, etc. Through interviews and questionnaires, Lei [18] found that the individual investors who are able to effectively master market information and have an advantage over others on investment knowledge will be more likely to profit. Some scholars studied the features of specific investment behaviors in excessive trading, and considered that those excessive trading behaviors are common among individual investors [19, 20]. Others undertook research on the personal characteristics of individual investors, arguing that Chinese individual investors not only have a cognitive behavioral deviation in general sense, but also have localization deviation [21].

With the establishment of behavioral finance theories, studies on investors’ personal characteristics and behavior preferences have drawn scholars’ attention from both developed and emerging economies. The literature mentioned above provides a solid foundation for this research. However, there are few studies from the viewpoint of big data applications such as precision marketing and personalized service. The following conclusions can be drawn from existing literature: (1) most of the studies focused on the effects of investment and examined the influence of investor’s personal and behavior characteristics as explanatory variables of models; (2) many personal characteristics were analyzed from the aspects of psychological cognition and character traits, which needs professional and complex psychological tests, tracking surveys, therefore being constrained to relatively small samples sizes; (3) most researches merely applied classical linear regression model for analysis, seldom using models of data mining which potentially reveal any nonlinear, discontinuous and probabilistic relationships between variables. As a result, we inferred that it is necessary to use a larger sample and more accurate methods to study the predictability of investor decision-making behaviors from the perspective of data applications.

## Variables and data

### Demographic characteristics variables

As mentioned in literature reviews, personal characteristics and behaviors of investors are extensive. However, many of them are hard to obtain, which would prevent them from being used as predictor variables of the models in practical business intelligence projects. For example, many business intelligence projects would face “cold start” problems when the project is at the starting stage, which usually represents a serious problem in recommender systems as there is not enough historical data to analyze user’s preferences at the beginning. And in the same way, there is insufficient data to build precise models. In this paper, a solution is provided by focusing on some demographic characteristics which are comparatively easy to acquire. Referring to research in [22–26], this paper focuses on the following demographic characteristics: gender, age, occupation, years of education, financial knowledge level, investment experience and income. Here we call them DC (Demographic Characteristic) variables. These characteristics are not only accessible in daily life, but also can be measured and described easily. Moreover, these characteristics are stable within a certain period of time. As input variables of models, these features are of great importance for practical applications.

### Investment behavior variables

Although investors would not deliberately pay attention to and structure their own behaviors, according to decision-making theories, they naturally or half unconsciously follow such processes composed of four stages: preparation, decision making, execution and feedback. The main tasks in the preparation stage include evaluating self-ability, and determining investment goals and searching information; in the decision-making stage, the most important tasks are choosing investment directions and products as well as determining investment scale and allocation proportions; the decision execution stage includes determining trading time and specific trading operations; and the feedback stage is to evaluate and rethink the previous decisions. Based on this viewpoint and referring to certain other available studies [27–33], this paper investigates following specific investment decision behaviors: investment scales, investment instruments, transaction frequencies, decision-making styles, investment information channels. All of these are major behaviors of investors in different decision stages and have potential value for financial marketing and service. Here we call them IB (Investment Behavior) variables.

### Survey sample overview

A questionnaire was designed in accordance with DC and IB variables. Some questions for measuring the validity and consistency of the questionnaire are also included. The questionnaire could be completed within about 15 minutes. According to *the Statistical Report of Development Status of China Internet Network* released by China Internet Network Information Centre (CNNIC), in December 2013, the number of Chinese cyber citizens have reached 618 million. Nowadays, the vast majority of investment transaction are handled through the network, hence most financial investors are cyber citizens too. Therefore, we hired a professional online survey company (https://www.wenjuan.com/) to issue questionnaires. This process consisted of two stages. The first stage started in December 2013 and ended in February 2014. The second stage lasted from November 2014 to December 2014. Altogether, around 22,000 questionnaires were collected. In the procedure of data pretreatment, we have removed those questionnaires from duplicate IP address or due to lack of validity or consistency. Moreover, the questionnaires with abnormal answer times or unanswered key questions were also rejected as invalid. Finally, we use 8,489 questionnaires as experimental data, the survey data were collected anonymously and the data can be found through the following URL: https://github.com/WennieX2017/IID-Behavior-Prediction.

Table 1 describes the geographical distribution of survey samples. The table illustrates that the samples are mainly from six developed provinces or municipalities namely Guangdong, Shanghai, Beijing, Shandong, Jiangsu, Zhejiang, which account for 55.92%. To some extent, the distribution also reflects current economic geography in China and can prove the geographical representativeness of the samples.

**Table 1:** Geographical distribution of survey objects.

Region	Percent	Region	Percent	Region	Percent
Guangdong	15.29%	Guangxi	3.08%	Yunnan	0.99%
Shanghai	11.06%	Tianjin	2.98%	Guizhou	0.52%
Shandong	8.25%	Anhui	2.87%	Xinjiang	0.47%
Beijing	7.60%	Liaoning	2.86%	Neimenggu	0.45%
Zhejiang	7.14%	Hunan	2.02%	Hainan	0.25%
Jiangsu	6.57%	Shanxi	1.88%	Gansu	0.22%
Hebei	4.38%	Chongqing	1.77%	Ningxia	0.15%
Hubei	3.57%	Jiangxi	1.76%	Qinghai	0.04%
Fujian	3.37%	Shanxi	1.52%	Unknown	0.22%
Sichuan	3.27%	Heilongjiang	1.18%	—	—
Henan	3.25%	Jilin	1.02%	—	—

**Fig 1:**
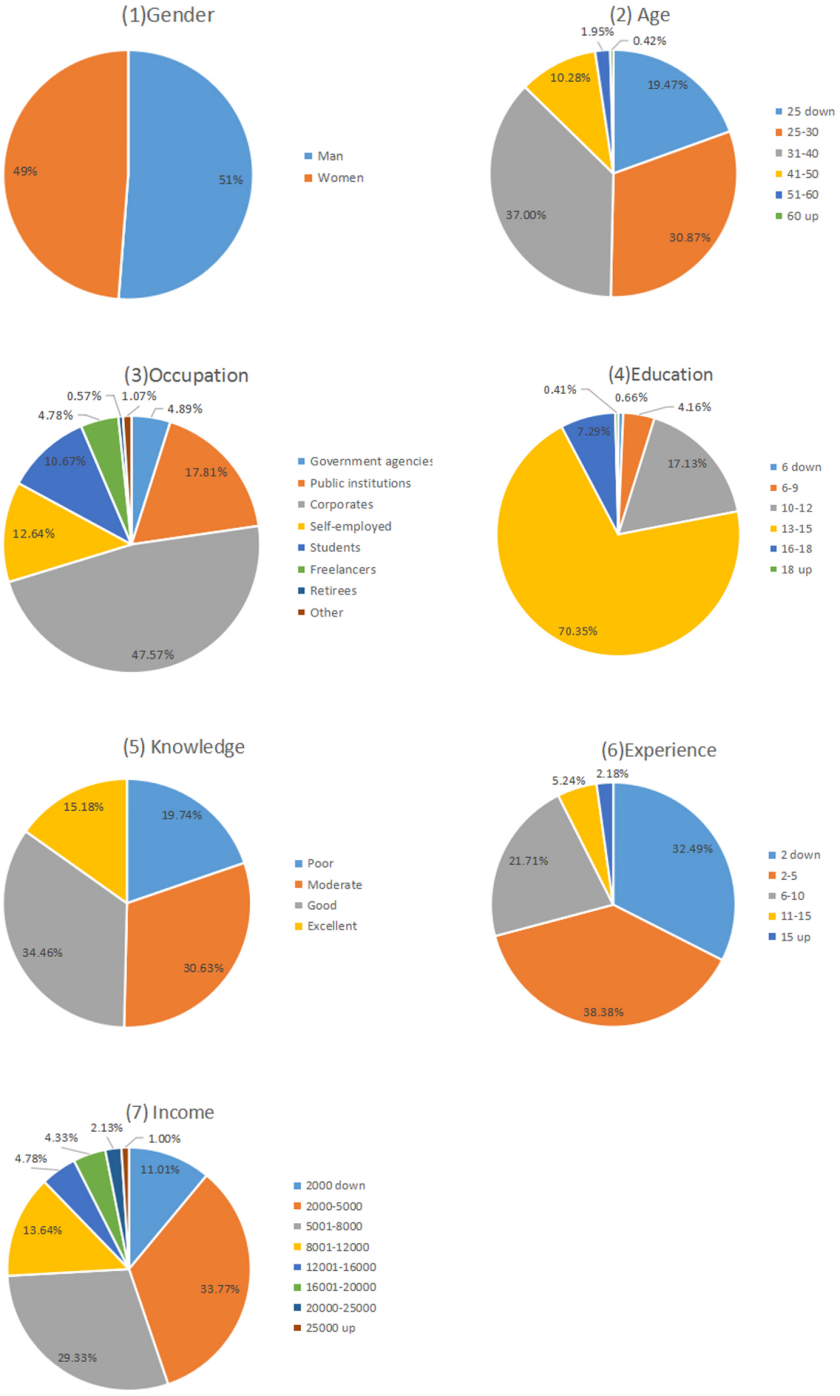
Distribution of demographic characteristic variables.

Fig 1 shows the distribution of three DC variables. We can find that the age, occupation and income structure of investors are very similar to that of the *China depository and clearing statistical yearbook (2015)* and the survey by China Fund Industry Association in 2013 and 2014.

**Fig 2:**
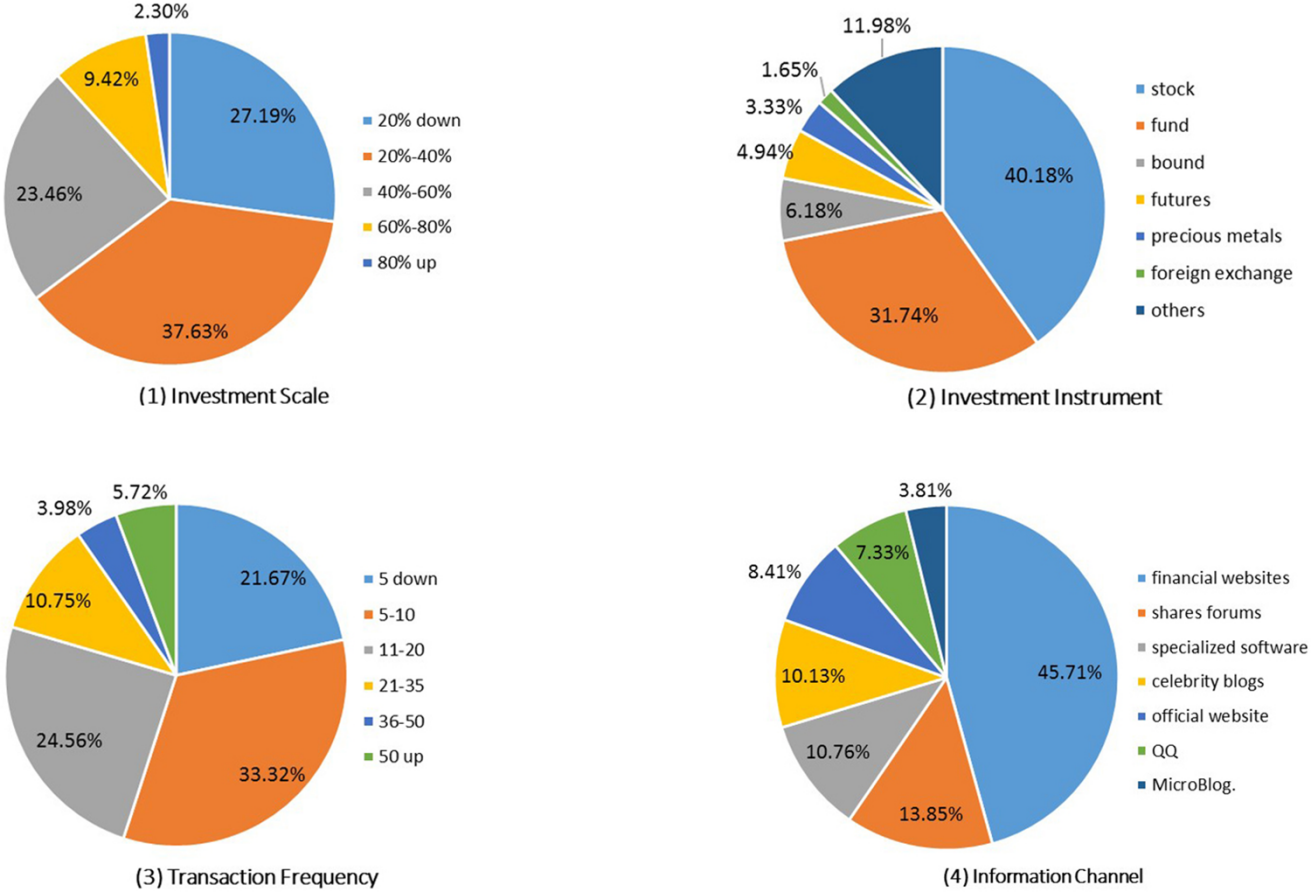
Distribution of investment behaviors.

Fig 2 presents the distributions of four IB variable values. It shows that investment scale is generally less than 40% of disposable assets; about half of investors made no more than 10 transactions per year, while about 20% active investors made more than 20 trades annually; approximately 70% of investors focus on traditional stock or fund investments; finance and economics websites are main sources for individual investors to acquire the investment decision information. These results are roughly consistent with *Shenzhen Stock Exchange 2013 Survey Report of Individual Investor Situations*.

Correlating results from other information sources, it is believed that the survey data are consistent with other information released by China’s official agencies in several ways. Consequently, it could be regarded as a representative sample of individual investors in China. In addition, some data preprocessing steps such as binning and reclassifying were executed. Table 2 displays the values of each variables for subsequent analyses.

**Table 2:** Variable description.

Variable		Variable value	description
**DC**	Gender	1.(Man) 2.(woman)	
	Age	1.(25 down) 2.(26–30) 3.(31–40) 4.(41–50) 5.(51–60) 6.(60up)	Years
	Occupation	1.(Government agencies) 2.(Public institutions) 3.(Corporates) 4.(Self-employed) 5.(Students) 6.(Freelancers) 7.(Retirees) 8.(Other)	
	Education	1.(6 down) 2.(6–9) 3.(10–12) 4.(13–15) 5.(16–18) 6.(18 up)	Years of Education
	Knowledge	1.(Poor) 2.(Moderate) 3.(Good) 4.(Excellent)	Investment knowledge&skill
	Experience	1.(2 down) 2.(2–5) 3.(6–10) 4.(11–15) 5.(15 up)	Years engaged in financial investment
	Income	1.(2000 down) 2.(2000–5000) 3.(5001–8000) 4.(8001–12000) 5.(12001–16000) 6.(16001–20000) 7.(20000–25000) 8.(25000 up)	Monthly income(Yuan)
**IB**	Investment Scale	1.(40% down) 2.(40% up)	The proportion of investment to disposable funds
	Investment instrument	1.(Stock) 2.(Other)	Most favorite investment instrument
	Transaction frequency	1.(Low(≤10)) 2.(High(>10))	Trade times per year
	Decision-making Style	1.(Decisive) 2.(Cautious)	
	Information Channel	1.(Financial website, Official website) 2.(Other)	

## Methodology

In order to ensure the accuracy of the results and considering the nonlinear, discontinuous and uncertain relationship among variables, Pearson’s chi-squared test, Spearman rank correlation test and several data mining methods are applied in subsequent analyses.

**Fig 3:**
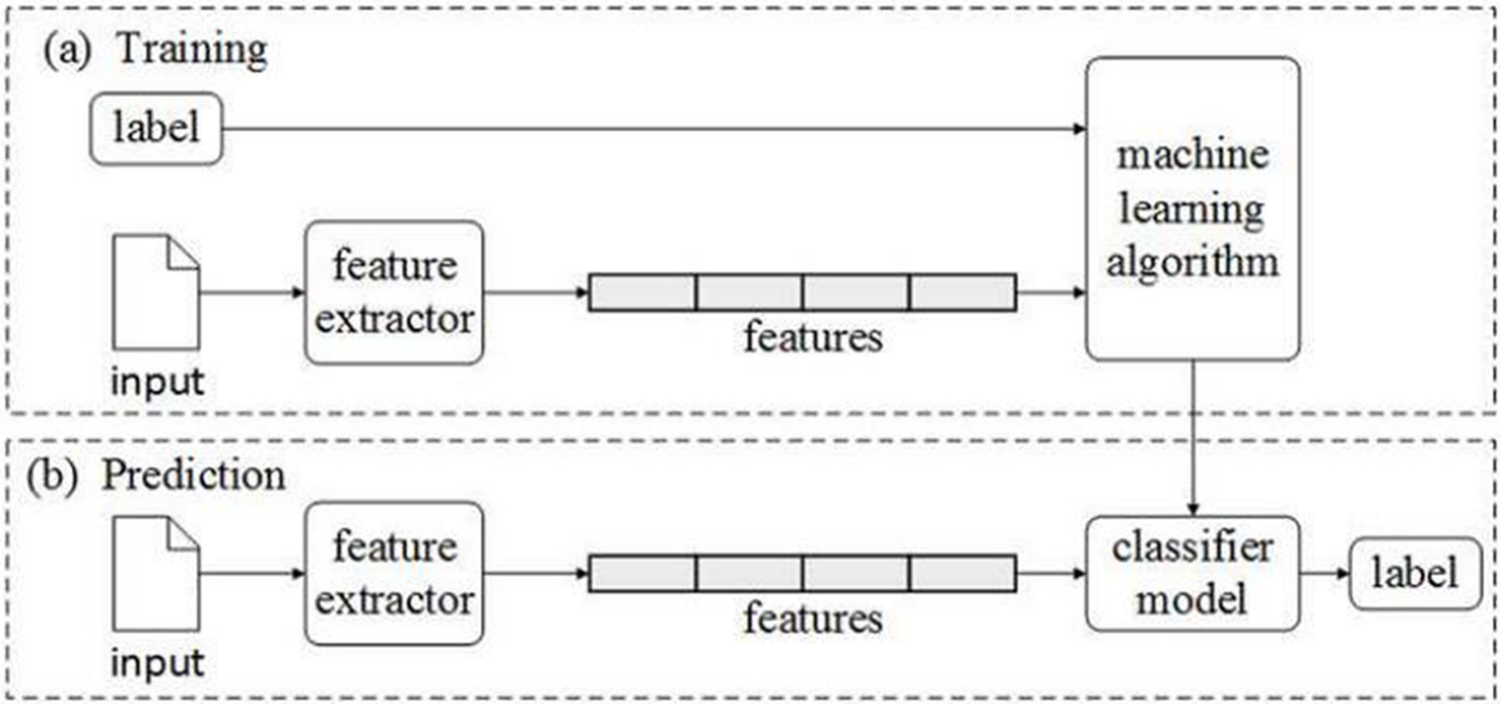
Supervised classification.

1. Pearson’s chi-squared test (*χ*^2^) is a statistical test suitable for unpaired data from large samples [34]. Its null hypothesis states that the frequency distribution of certain events observed in a sample is consistent with a particular theoretical distribution. The value of the test-statistic is:
  $$\chi^{2}=\sum_{i=1}^{n}\frac{{\left(O_{i}-E_{i}\right)}^{2}}{E_{i}}=N\sum_{i=1}^{n}\frac{{\left(O_{i}/N-p_{i}\right)}^{2}}{p_{i}}$$
  Where *χ*^2^ = Pearson’s cumulativetest statistic, which asymptotically approaches a *χ*^2^ distribution.
  *O*_*i*_ = the number of observations of type i.
  N = total number of observations
  *E*_*i*_ = N* *p*_*i*_ = the expected (theoretical) frequency of type i, asserted by the null hypothesis that the fraction of type i in the population is *p*_*i*_.
  n = number of categories.
  The chi-squared statistic can then be used to calculate a p-value by comparing the value of the statistic to a chi-squared distribution. If the test statistic exceeds the critical value, the null hypothesis (there is no difference between the distributions) can be rejected. Here, based on two stages of data collection, we tested each pair of variables between DC and IB. If the null hypothesis is rejected, then it means the DC variable has information to predict the IB variable, because various value of the DC variable matches to different distribution of the IB variable.
2. Since the variables such as age, education and transaction frequency are ordinal variables, Spearman Rank Correlation method is applied; this is a nonparametric (distribution-free) rank statistical analysis tool proposed by Charles Spearman. It assesses how well an arbitrary monotonic function can describe a relationship between two variables, without making any assumptions about the frequency distribution of the variables. It does not require the assumption that the relationship between the variables is linear, nor does it require the variables to be measured on interval scales; it is very suitable for variables measured at the ordinal level. Spearman correlation coefficient can be computed using the following formula.
  $$\rho =\frac{Cov(x,y)}{\sigma_{x}\sigma_{y}}$$
  Where *Cov*(*x*, *y*) is the covariance of the rank variables x and y, and *σ*_*x*_, *σ*_*y*_ are the standard deviations of the rank variables. We would analyze the correlation of each pair of ordinal variables between DC and IB at two stages of data collection. If the correlation coefficient ρ is significantly different from 0, it means the DC variable has information to predict the IB variable.
3. Above Pearson’s chi-squared test and Spearman Rank Correlation analysis (described above) merely investigate the correlation between one DC variable and one IB variable each time. However, investors’ behavior is comprehensively affected by multiple factors (variables). In order to test the predictability of each IB variable based on combination of different DC variables, data mining is applied. Generally, data mining is based on inductive statistics and is a type of data driven method, which is especially suitable for finding the hidden, complex nonlinear models of the data [35]. Supervised classification is the most important data mining technology, whose framework is shown in Fig 3. It usually includes a training and a test (prediction) process. During training, pairs of feature sets and labels are fed into the machine learning algorithm to generate a model. During prediction, these feature sets are then fed into the model to generate predicted labels. Here, six classic and widely used machine learning algorithms: C4.5 (decision-making tree), C&R (Classification and Regression Tree), BP (Back Propagation Neural Network), SVM (Supportive Vector Machine), LR (Logistic Regression) and NB (Naive Bayes) are carried out. For detailed principles about these data mining algorithms, please see reference [36]. Here, the data collected from the first stage are used as a training set, and those from the second stage are used as a test (prediction) set. Through this we can not only view the predictive effect, but also understand the stability of the models in different periods.

For classification tasks, Precision, Recall, F-measure and Accuracy are widely used to evaluate the performance of models. To understand them, let us consider a two-class prediction problem, in which the outcomes are labeled either as positive (p) or negative (n). There are four possible outcomes from the classifier. If the outcome is p and the actual value is also p, then it is called a true positive (tp); however, if the actual value is n then it is a false positive (fp). Conversely, a true negative (tn) means both the prediction outcome and the actual value are n, and false negative (fn) represents the prediction outcome is n but the actual value is p (see Table 3).

**Table 3:** Confusion matrix.

		Prediction Value	
		Positive	Negative
True Value	Positive	true positive (tp)	false negative (fn)
	Negative	false positive (fp)	true negative (tn)

Accuracy (Accu) is the ratio between the number of correctly predicted samples and the number of the total samples, which is defined as:

$$Accu=\frac{tp+tn}{tp+fn+fp+tn}$$

Precision (P) is the ratio between the number of true positive and the total number of positive predicted by the classifier, which is defined as

$$P=\frac{tp}{tp+fp}.$$

Recall (R) is the ratio between the number of detected positive and the total number of positive that occurred during a classification, defined as

$$R=\frac{tp}{tp+fn}.$$

F-measure combines precision and recall which is defined as the harmonic mean of precision and recall.

$$F=\frac{2PR}{P+R}$$

Value range of above three indicators are all between 0 and 1. The higher of them, the model prediction will perform better.

## Analysis results

### Results of Pearson’s chi-squared test

Table 4 presents the results of Pearson’s chi-squared test for each pair of DC-IB variables in two stages of data collection.

**Table 4:** Results of Pearson’s chi-squared test.

		Scale	Instrument	Frequency	Style	Channel
**Gender**	Stag1	15.00***	43.68***	82.51***	13.54***	0.09
	Stag2	25.72***	87.10***	111.33***	6.58**	2.26
**Age**	Stag1	101.63***	215.93***	286.11***	56.72***	6.68
	Stag2	91.93***	241.60***	338.43***	76.02***	16.51***
**Occupation**	Stag1	102.28***	102.23***	187.92***	17.55**	12.05*
	Stag2	80.88***	72.19***	198.83***	9.39	24.48***
**Education**	Stag1	5.86	19.37***	32.84***	11.88**	11.82**
	Stag2	13.20**	29.04***	66.85***	25.25***	11.30**
**Knowledge**	Stag1	188.22***	173.23***	300.43***	64.79***	11.04**
	Stag2	189.52***	136.19***	263.11***	80.27***	7.58*
**Experience**	Stag1	298.37***	467.21***	725.21***	40.12***	14.32***
	Stag2	337.71***	476.05***	965.79***	29.16***	14.66***
**Income**	Stag1	219.02***	228.98***	576.24***	66.15***	11.34
	Stag2	270.12***	251.87***	585.49***	49.31***	9.64

Note: the chi-squared statistic is marked with ** when it’s significant at level of 0.05 and marked with *** at level of 0.01.

According to the results, most IB variables are significantly associated with DC variables at level of 0.01, which means that the DC variables contain information to predict the IB variables. For example, the result shows that gender is significantly related to investment frequency, which is consistent with the previous research finding that “men were more likely than women to adjust their investments” [12]. Besides, the significant correlation between gender and investment scale also matches Barber and Odean’s finding that “women hold slightly, but not dramatically smaller, common stock portfolios” [37]. In the meantime, the Chi-squared values of each DC-IB variables pair at two stages are very similar, which indicate that the relationships between them are stable.

### Results of rank correlation analysis

As shown in Table 5, most Spearman Rank Correlation coefficients are significant at the level of 1%, which indicates that these ordinal DC variables generally have a significant impact on the IB variables. To be specific, age, education, knowledge, experience, and income are all positive with investment scale, that is consistent with the general perception that with the growth of investors’ experience, knowledge, age and income, investors often have better investment capability and consciousness. In addition, the significant positive relationship between transaction frequency and experience is consistent with the research conclusion that “experienced investors were generally over-confident, thus leading to frequent trading” [21]. And the decision-making style and age present a negative correlation, reflecting that the older investors are more inclined to have a cautious decision-making style, which is consistent with the intuitive feel that older people are more conservative. Overall, in two stages, the correlation coefficients of most pairs of ordinal variables are very close and have the same sign (positive or negative), which indicate that these relationships are stable.

**Table 5:** Spearman rank correlation coefficients.

		Scale	Frequency	Style
**Age**	Stag1	0.091***	0.234***	-0.088***
	Stag2	0.086***	0.233***	-0.087***
**Education**	Stag1	0.020***	0.091***	0.044***
	Stag2	0.038***	0.112***	0.056***
**Knowledge**	Stag1	0.195***	0.248***	-0.048***
	Stag2	0.184***	0.175***	0.031**
**Experience**	Stag1	0.281***	0.441***	-0.054***
	Stag2	0.265***	0.447***	0.004
**Income**	Stag1	0.232***	0.394***	-0.019
	Stag2	0.230***	0.346***	0.013***

Note: the correlation coefficients marked with ** when it’s significant at level of 0.05 and marked with *** at level of 0.01.

### Results of data mining models

We created our predictive models using IBM SPSS Modeler Version 18.0. SPSS Modeler is a predictive analytic software that provides a range of advanced algorithms and techniques for data analysis, decision management and optimization.

Table 6 indicates the predictive performance of the models on test (stage 2) data set. The accuracy of applying demographic characteristics to predict investment behavior in different classification methods has reached a good level (far more than 0.5). At the same time, most of the values of R, P and F are over 0.5 too, which indicate that these models are strong predictors. Even though certain values of R, P and F are not high, the corresponding model possess utility and value: for instance, applying the C&R model to the prediction of investment style, the R, P and F values are 0.49, 0.36 and 0.42 when style = 1, while when style = 2, the values of the R, P and F are 0.70, 0.80 and 0.74. It means that although this model is not suitable for predicting style = 1, but would be very good at predicting style = 2. Hence the model is still valuable for finding clients with style = 2.

**Table 6:** Results of data mining models.

		Style			Instrument			frequency			Channel			Scale		
		1	2	Accu	1	2	Accu	1	2	Accu	1	2	Accu	1	2	Accu
**C4.5**	**R**	0.45	0.69	0.63	0.68	0.63	0.65	0.67	0.72	0.70	0.62	0.48	0.56	0.58	0.67	0.61
	**P**	0.34	0.78		0.51	0.77		0.73	0.66		0.62	0.48		0.76	0.47	
	**F**	0.39	0.73		0.59	0.69		0.70	0.69		0.62	0.48		0.66	0.55	
**C&R**	**R**	0.49	0.70	0.64	0.66	0.63	0.65	0.68	0.74	0.71	0.60	0.48	0.55	0.63	0.63	0.65
	**P**	0.36	0.80		0.51	0.76		0.75	0.67		0.61	0.47		0.75	0.49	
	**F**	0.42	0.74		0.58	0.69		0.72	0.70		0.61	0.47		0.69	0.55	
**SVM**	**R**	0.48	0.68	0.63	0.67	0.66	0.66	0.71	0.68	0.69	0.57	0.53	0.55	0.66	0.62	0.64
	**P**	0.35	0.79		0.53	0.78		0.72	0.67		0.62	0.47		0.75	0.50	
	**F**	0.40	0.73		0.59	0.71		0.71	0.67		0.59	0.50		0.70	0.55	
**BP**	**R**	0.57	0.58	0.58	0.67	0.63	0.65	0.66	0.76	0.71	0.56	0.47	0.52	0.64	0.62	0.63
	**P**	0.32	0.79		0.51	0.77		0.76	0.66		0.59	0.44		0.75	0.49	
	**F**	0.41	0.67		0.58	0.69		0.70	0.71		0.57	0.45		0.69	0.55	
**NB**	**R**	0.53	0.61	0.63	0.69	0.62	0.65	0.70	0.71	0.71	0.58	0.48	0.54	0.61	0.65	0.62
	**P**	0.55	0.59		0.51	0.78		0.74	0.67		0.60	0.45		0.76	0.48	
	**F**	0.54	0.60		0.59	0.69		0.72	0.69		0.59	0.47		0.67	0.55	
**LR**	**R**	0.54	0.61	0.59	0.69	0.64	0.66	0.68	0.73	0.71	0.63	0.47	0.56	0.62	0.66	0.63
	**P**	0.33	0.79		0.52	0.78		0.74	0.67		0.62	0.48		0.76	0.49	
	**F**	0.41	0.69		0.60	0.70		0.71	0.70		0.62	0.47		0.68	0.56	

Comparing the results of different models relevant to IB variables, the best performing model is for transaction frequencies, where the predictive accuracy of all six classifiers has reached around 0.7, followed by investment instruments and investment scales. Therefore, demographic characteristics possess strong predictive capability to the investors’ behaviors.

Comparing the performance of six classifiers, the C&R method appears to give the best results on almost every IB variables, thus, it could be recommended as a method for building a predictive model. Moreover, the C&R method has some unique advantages. For instance, it can output the degree of importance of predictor variables to the goal variable, and produce decision rules like “if …then…”.

Table 7 demonstrates the importance of DC variables to each IB variable in the C&R model. The degree of importance represents the DC variables’ relative contribution to the prediction of the goal variable (showed in the brace).

**Table 7:** Importance of DC to IB variables.

IB variable	Importance of DC variables
**Scale**	Experience(0.49) > Income(0.21) > Occupation(0.10) > Age(0.05)≥Gender(0.05) ≥ Knowledge(0.05) ≥ Education(0.05)
**Instrument**	Experience(0.59) > Income(0.13) > Occupation(0.09) > Age(0.08) > Gender(0.05) > Education(0.04) > Knowledge(0.02)
**frequency**	Experience(0.59) > Income(0.20) > Knowledge(0.06) > Age(0.05) > Gender(0.04) > Education(0.04) ≥ Occupation(0.03)
**Style**	Knowledge(0.24) > Income(0.20) > Age(0.16) > Education(0.13) > Occupation(0.09) ≥ Gender(0.09) > Experience(0.08)
**Channel**	Knowledge(0.25) > Experience(0.22) > Occupation(0.12) ≥ Income(0.12) > Age(0.11) ≥ Gender(0.11) > Education(0.07)

Based on the results, it is obvious that there are strong connections between all the DC variables and IB variables. In addition, the importance of variables provides more information in details. For example, the investment scales of individual investors have the strongest link with their investment experiences, incomes and occupations. Investment style is mainly influenced by knowledge, income, age and education background. Investment instrument and trade frequency are dominated by experience and income. And financial knowledge and investment experience are the major factors of information channel. These results are in agreement with the practical experiences and observations. From these results, we can conclude that experience and income are most important factors for nearly all behaviors. The importance of knowledge, occupation and age are significantly different for behaviors.

Table 8 illustrates several decision rules with high confidence values learned with C&R. From these rules, we can conclude that experienced investors with high income are more likely to take risks (scale>40%) (No. 2); those of ages from 50 to 60 with limited investment knowledge are inclined to make decisive decisions (No.7); those who are experienced, with high income and sufficient investment knowledge are usually high frequency trading players (No.6). Obviously, these rules are in accordance with investors’ behaviors and are valuable for further applications.

**Table 8:** Instances of predictive rule from C&R.

IB variable	No.	Rule	Confidence
**Scale**	1	**if** (experience = 1) **then** scale = 1	0.68
	2	**if** (experience≥3) **and** (income≥3) **then** scale = 2	0.69
**Instrument**	3	**if** (experience≥3) **and** (gender = 1) **and** (age≥4) **then** instrument = 1	0.81
	4	**if** ((occupation = 1) **or** (occupation = 6) **or** (occupation = 8)) **and** (experience = 1) **and** ((income≤2) **or** (income≥6)) **and** ((knowledge≤2)) **then** instrument = 2	0.90
**Frequency**	5	**if** (experience = 1) **and** ((income = 1) **or** (income = 2)) **then** frequency = 1	0.83
	6	**if** (experience≥3) **and** (income≥4) **and** (knowledge≥3) **then** frequency = 2	0.86
**Style**	7	**if** (age = 5) **and** (knowledge = 1) **then** style = 1	0.83
	8	**if** (age = 1) **and** ((knowledge = 2) **or** (knowledge = 3)) **and** ((income = 2) **or** (income = 3) **or** (income = 4)) **then** style = 2	0.70
**Channel**	9	**if** ((occupation = 1) **or** (occupation = 3) **or** (occupation = 4)) **and** ((experience = 2) **or** (experience = 3) **or** (experience = 4)) **and (**(income = 2) **or** (income = 5) **or** (income = 8)) **and** (age = 2)) **then** channel = 1	0.66
	10	**if** ((occupation = 2) **or** (occupation = 5) **or** (occupation = 6) **or** (occupation = 8)) **and** ((experience = 2) **or** (experience = 3) **or** (experience = 4)) **and** ((income = 1) **or** (income = 3) **or** (income≥5)) **and** (age = 2) **then** channel = 2	0.75

## Application case

In order to prove the usefulness of the findings above, we assume that a financial institution is going to promote a particular financial service to clients with a certain behavioral preference, for example, risk-preference investors (scale more than 40%). This institution keeps a list of 100,000 clients with the above demographic characteristics, but without any risk-preference information. Let us assume that the promotion costs are 10 RMB per person, each customer who buys this service (potential responder) would bring 250 RMB revenue. Response rate of risk-preference investors is 10%, while the rate for non-risk-preference investors is 1%. Here a decision must be made: should all potential responders be targeted or just some of them?

When promoting to all potential customers, it would reach all risk-preference clients whose number is about 35,180 (risk-preference investors account for about 35.18%). Suppose the institution releases questionnaires and collects data according to this research, and got a predictive rule like No.2 in Table 9 from data mining model. According to this rule, the institution would only target experienced(≥3) and high income(≥3) investors, which covers around 25% of total investors, and reach about 17,250 (25,000×69%) risk-preference investors. Table 9 compares the benefits of this promotion when using this prediction model and when not.

**Table 9:** Result of benefits using different marketing way.

	Marketing without prediction	Marketing with prediction
Target Number	100,000	25,000
Promotion Cost	1,000,000	250,000
Number of Responders	35,180×10% + 64,820×1% = 4,166	17,250×10%+7,750×1% = 1,802
Revenue	4,166×250 = 1,041,500	1,802×250 = 450,500
Modeling Cost	0	40,000
Profit	41,500	160,500

Based on these results, this financial service institution can earn a profit (160,500 RMB) even considered modeling cost for precision promotion activity (40,000 RMB), which is better than the solution of using model (41,500 RMB).

## Conclusions

Understanding the behaviors of investors is of great value to many financial institutions. However, the investors’ behavior information is ambiguous and implicit, which makes it difficult to observe, measure and obtain directly. This paper presents a new idea: analyze the capability to predict investment behaviors based on certain demographic characteristics, and verify the feasibility and effectiveness of building behavior prediction models based on these characteristics. Our study makes the following contributions:

1. Different from that most studies focused on the effects of investment and examined the influence of investor’s demographic and behavior characteristics as explanatory variables of models. We explored the potential relationship between investor’s demographic characteristics and investment behaviors, and proved that investors’ demographic characteristics can be used to predict their investment behaviors.
2. We apply certain easy-to-obtain personal characteristics as predictors to build models to evaluate investors’ behavior preferences. In this way, the issue that investors’ behavior preferences are hard to obtain and measure due to their dynamics, ambiguity, heterogeneity and uncertainty can be solved.
3. We use data mining which can reveal nonlinear, discontinuous and probabilistic relationships between variables to study the predictability of investor decision-making behaviors from the perspective of data applications.

In this paper, an in-depth study about the predictive power of several easy-to-obtain demographic characteristic variables on investors’ behaviors has been conducted and following conclusions drawn:

1. By applying Pearson’s chi-squared test, Spearman rank correlation analysis, and six classic data mining techniques, we can find that Chinese investors’ decision behaviors are significantly and stably correlated to their demographic characteristics, which indicates that the demographic characteristics can be used for prediction of investors’ behaviors;
2. Among the demographic variables examined in this paper, experience and income are especially important predictors. And trade frequency of an investor is the most predictable behavior, followed by investment scale and investment instrument;
3. Due to the availability of demographic characteristics, it is an economical and feasible approach for predicting investors’ behaviors. Even if investors’ behaviors cannot be predicted exactly, information hidden in demographic characteristics is still valuable for some applications such as precision marketing, personalized service and so on. Especially at the starting phase, data on demographic characteristics can be useful supplements when behavioral data are insufficient to address the “cold start” problem of business intelligence projects.

## Supporting information

S1 File Questionnaire of individual investors. (DOC)

S2 File Questionnaire of individual investors_Chinese version. (DOC)
