# Supplementary material for: Individual investment decision behaviors based on demographic characteristics: Case from China
Source: PLoS One. 2018 Aug 9;13(8):e0201916. doi: 10.1371/journal.pone.0201916 (PMC6085059; doi:10.1371/journal.pone.0201916)
Supplement: S1 File — (DOC) [file pone.0201916.s001.doc]

# Questionaire of Individual Investors

| Dear friends, I would like to thank you for taking the time to fill in this questionnaire. This survey is tend to explore the relationship between information media and your investment habits through systematic and objective analysis, and to understand the impact of information media on your decisions making.  The main purpose of this questionnaire is to collect data for academic research. It does not involve personal privacy. The information collected will be used in pure academic research instead of being used for any commercial purpose. Anonymous questionnaires are adopted to protect your privacy. Please fill in it at ease.  This questionnaire will take up about 10 minutes. Thank you for your help again. |
| --- |
| **Please fill out the essential information. We promise to keep the information secret.** |
|  |
| **1.What is your gender?** [Single Choice] [Required] |
| ○ male    ○ female |
|  |
| **2.What is your current age?** [Single Choice] [Required] |
| | ○ 25 down | ○ 26~30 | ○ 31~40 | ○ 41~50 | ○ 51~60 | ○ 60 up |  | | --- | --- | --- | --- | --- | --- | --- | |
|  |
| **3.What is your occupation?** [Single Choice] [Required] |
| ○ Students    ○ Government agencies    ○ Public institutions    ○ Company employee    ○ Self-employed    ○ Freelancers    ○ Retirees    ○ Other |
|  |
| **4.what is your background?** [Single Choice] [Required] |
| ○ Junior high school    ○ Senior high school    ○ Vocational school    ○ Undergraduate college    ○ Self-employed    ○ Master |
|  |
| **5.How much do you know about investment knowledge?** [Single Choice] [Required] |
| ○ Major for finance and economics major and has a systematic knowledge system.     ○ Have a self-study of investment knowledge and read more than five books about investment.    ○ Have a self-study of investment knowledge and read less than five books about investment.    ○ Have never learned investment knowledge and has hardly read related books. |
|  |
| 1. **How many years of investment experience do you have?** [Single Choice] [Required] |
| ○ < 2    ○ 2 ~ 5    ○ 6 ~ 10    ○ 11 ~ 15    ○ > 15 |
|  |
| **7.Your monthly income is about?** [Single Choice] [Required] |
| | ○ 2000 down | ○ 2000~5000 | ○ 5001~8000 | ○ 8001~12000 |  | | --- | --- | --- | --- | --- | | ○ 12001~16000 | ○ 16001~20000 | ○ 20001~25000 | ○ 25000 up |  | |
|  |
| | 1. **What's the percent of your total household assets are spent on investment?**   [Please enter a number from 1 to 100] [Required] | | --- | |
|  |
| **9.What are the investment instruments you have used?** [Multiple Choice] [Required] |
| □ Stock    □ Future    □ Bond    □ Fund    □ Foreign exchange    □ Noble metal    □ Bank’s investment banking products which are not capital protected     □ Online investment products     □ Other _________________ |
|  |
| **10.What is the investment instrument you mainly use**? [Single Choice] [Required] |
| ○ Stock    ○ Future    ○ Bond    ○ Fund    ○ Foreign exchange    ○ Noble metal    ○ Other _________________ * |
|  |
| **Please answer the following questions according to your actual situation in the last year or two.** |
| 1. **What is your average number of transactions per year is about?**   [Single Choice] [Required] |
| ○ < 5    ○ 5 ~ 10    ○ 11 ~ 20    ○ 21 ~ 35    ○ 36 ~ 50    ○ > 50 |
|  |
| **12.What kind of behavior do you usually have on the Internet?**: [Single Choice] [Required] |
| ○ Expressing dissatisfaction after suffering a loss    ○ Expressing joy after obtaining the expected return    ○ None of the above two behaviors |
|  |
| **13.What kind of speech do you usually make on the Internet?** [Single Choice] [Required] |
| ○ A view that is inconsistent with the current mainstream.    ○ A view that is consistent with the current mainstream.    ○ None of the above two statements |
|  |
| 1. **Will you publish or forward the information that you think is useful?**   [Single Choice] [Required] |
| ○ Never    ○ Seldom    ○ Often |
|  |
| 1. **How many times do you usually publish or forward the same information that you think is useful?**（Please fill in the numbers）[Required] |
| _________________________________ |
|  |
| 1. **How long do you usually spend to identify the authenticity of network information and make investment decisions?** [Single Choice] [Required] |
| ○ Make a judgement by intuition for a few minutes    ○ Contact with other information and think about it for one to two hours    ○ It will take more than 2 hours to check and verify each item one by one. |
|  |
| | 1. **When someone in your frequently used information channel recommends "insider information" to you secretly, how far can you trust them ?** [Please enter a number from 1 to 100] [Required] | | --- | |
|  |
| 1. **When there are many public opinions with obvious bias on the Internet,will you change the investment decisions you have made ?** [Single Choice] [Required] |
| ○ I will change    ○ I will not change |
|  |
| **19.When you make investment decisions, is the market consistent with the direction of the previous opinion ?** [Single Choice] [Required] |
| ○ In most cases, the market is consistent with previous public opinion    ○ I'm not sure about the relationship between them    ○ In most cases, the market is inconsistent with previous public opinion |
|  |
| **20.When you have decided to invest, you find that certain Internet comments doubt its potential. What kind of actions have you taken?** [Single Choice] [Required] |
| ○ Making comments on the Internet and recommending your own decision    ○ Look for the same comments as your own decision on the Internet    ○ Stick to your own decisions    ○ Give up your decision to avoid loss and fear.    ○ None of the above behaviors |
|  |
| **21.In what way do you usually get investment information?** [Single Choice] [Required] |
| ○ Newspapers and magazines    ○ Television program    ○  Internet media    ○ Acquaintance |
|  |
| **22.Where do you usually browse investment information ?** [Multiple Choice] [Required] |
| □ Stock BBS    □ Celebrity blog    □ Financial websites     □ QQ    □ Professional software     □ Micro-blog    □ Company websites |
|  |
| **23.In the following options, which is the most important information channel to your decision making?** [Single Choice] [Required] |
| ○ Stock BBS    ○ Celebrity blog    ○ Financial websites    ○ QQ    ○ Professional software    ○ Micro-blog    ○ Company websites |
|  |
| **24.Which block do you pay most attention to in above channels?**  [Single Choice] [Required] |
| ○ Earnings reports    ○ Acquaintance    ○ Stock review and analysis    ○ Revelations from net friends    ○ News bulletin    ○ Other |
|  |
| **25.Have you made a profit for the last three years?** [Single Choice] [Required] |
| ○ Yes    ○ No |
|  |
| 1. **Do you think your investment income has met your expectations?**   [Single Choice] [Required] |
| ○ Yes    ○ No |
|  |
| | 1. **When the results are not as expected, how much do you think the negative impact of external media information is on you?** [Please enter a number from 1 to 100] [Required] | | --- | |
|  |
| | **28.When the results are not as expected, how much do you think the negative impact of external media information is on you?**[Please enter a number from 1 to 100] [Required] | | --- | |
|  |
| **You have completed the full content of this survey.**  **Thank you again for your cooperation and support for our investigation. I wish you a brilliant success in your future investment.** |
