# Supplementary material for: Individual investment decision behaviors based on demographic characteristics: Case from China
Source: PLoS One. 2018 Aug 9;13(8):e0201916. doi: 10.1371/journal.pone.0201916 (PMC6085059; doi:10.1371/journal.pone.0201916)
Supplement: S2 File — (DOC) [file pone.0201916.s002.doc]

# 个体投资者情况的调查问卷

| 尊敬的女士/先生：       您好！首先十分感谢您能在百忙之中抽出宝贵的时间来填写此问卷。这次问卷调查希望通过系统、客观的分析研究，探索信息媒体与您投资习惯的关系，并了解信息媒体对您做出的决策的影响程度。       本问卷的主要目的是为了学术研究收集数据，不涉及个人隐私，所搜集的信息均用于纯粹学术研究，绝不用于任何商业目的，采取匿名问卷的形式，对您的信息承诺予以保密，请您放心填写。      本次问卷将占用您10分钟左右的宝贵时间。祝您答题愉快！ |
| --- |
| **请填写您的基本信息，对您填写的信息承诺予以保密！** |
|  |
| **1.**您的性别： [单选题] [必答题] |
| ○ 男    ○ 女 |
|  |
| **2.**您的年龄段： [单选题] [必答题] |
| | ○ 25以下 | ○ 26~30 | ○ 31~40 | ○ 41~50 | ○ 51~60 | ○ 60以上 |  | | --- | --- | --- | --- | --- | --- | --- | |
|  |
| **3.**您的职业： [单选题] [必答题] |
| ○ 全日制学生    ○ 政府单位人员    ○ 事业单位人员    ○ 公司企业人员    ○ 私营、个体工商业人员    ○ 自由职业者    ○ 离退休人员    ○ 其他 |
|  |
| **4.**您的学历： [单选题] [必答题] |
| | ○ 初中 | ○ 高中 | ○ 专科 | ○ 大学本科 | ○ 硕士研究生 | ○ 博士研究生 |  | | --- | --- | --- | --- | --- | --- | --- | |
|  |
| **5.**您对投资知识的了解程度： [单选题] [必答题] |
| ○ 财经类专业毕业，有系统的知识体系    ○ 自学的投资知识，并读过5本及5本以上的投资相关书籍    ○ 自学投资知识，只读过不到5本的投资相关书籍    ○ 基本没有学过投资知识，几乎没读过相关书籍 |
|  |
| **6.**您从事投资的年限有： [单选题] [必答题] |
| ○ 不足2年    ○ 2年至5年    ○ 6年至10年    ○ 11年至15年    ○ 15年以上 |
|  |
| **7.**您目前的月收入： [单选题] [必答题] |
| | ○ 2000以下 | ○ 2000~5000 | ○ 5001~8000 | ○ 8001~12000 |  | | --- | --- | --- | --- | --- | | ○ 12001~16000 | ○ 16001~20000 | ○ 20001~25000 | ○ 25000以上 |  | |
|  |
| | 1. 您愿意用于投资的资金占自己家庭总资产的比例约为多少：   [输入0(0%)到100(100%)的数字] [必答题] | | --- | |
|  |
| **9.**您已经有过投资的领域有： [多选题] [必答题] |
| □ 股票    □ 期货    □ 债券    □ 基金    □ 外汇    □ 贵金属    □ 银行风险理财产品（特指非保本型银行投资理财产品）     □ 互联网理财产品（如：人人贷之类的互联网P2P平台投资等）     □ 其他 _________________ |
|  |
| **10.**上一题中您最主要的投资领域是： [单选题] [必答题] |
| ○ 股票    ○ 期货    ○ 债券    ○ 基金    ○ 外汇    ○ 贵金属    ○ 其他 _________________ *  包括“银行风险理财产品”、“互联网理财产品“等 |
|  |
| **请根据您最近1~2年内的实际情况回答以下问题！** |
| **11.**您每年的平均交易次数约为： [单选题] [必答题] |
| ○ 不足5次    ○ 5次至10次    ○ 11次至20次    ○ 21次至35次    ○ 36次至50次    ○ 超过50次 |
|  |
| **12.**您平时在网络上有过哪种言论行为居多： [单选题] [必答题] |
| ○ 在受到了损失之后表达不满    ○ 在达到预期收益后分享喜悦    ○ 没有在网络上有过以上言论 |
|  |
| **13.**您平时在网络的哪种类型的言论居多： [单选题] [必答题] |
| ○ 发表与当前主流不一致的观点    ○ 发表与当前主流一致的观点    ○ 从没在网络上有过以上言论 |
|  |
| **14.**对于您认为有用的信息您会发布或转发吗： [单选题] [必答题] |
| ○ 从不转发    ○ 偶尔转发    ○ 经常转发 |
|  |
| 1. 对同一条您认为有用的信息，一般会发布或转发多少次：（请仅填写数字）   [填空题] [必答题] |
| _________________________________ |
|  |
| 1. 您通常会花多长时间来鉴别网络信息的真伪并做出投资决策：   [单选题] [必答题] |
| ○ 凭直觉第一时间就做出判断，几分钟就能搞定    ○ 联系其他信息稍微进行考虑，一般会累计思考1、2个小时    ○ 逐项进行核实进行反复思考，时间可能得花2小时以上 |
|  |
| | 1. 在您常用的信息渠道中有人私下向你推荐“内幕消息”时，您对其的置信度约   为多少： [输入0(0%)到100(100%)的数字] [必答题] | | --- | |
|  |
| **18.**当出现较多的有明显偏向的网络舆论时，是否会使您改变已做出的投资决策： [单选题] [必答题] |
| ○ 会改变，舆论倒向的一边多少会有他的道理    ○ 不会改变，网络舆论的真假难辨用意不明 |
|  |
| **19.**您的大多数投资决策做出后的市场行情是否和之前的网络舆论偏向相一致： [单选题] [必答题] |
| ○ 大多数行情与之前的舆论一致    ○ 不清楚它们的关系    ○ 大多数行情与之前的舆论不一致 |
|  |
| **20.**当您已经决定进行某一项投资时，发现有网络言论对其并不看好，你有过以下哪种举动： [单选题] [必答题] |
| ○ 上网发表言论推荐自己的决策    ○ 寻找网上与自己决策相同的言论    ○ 不去管它一直坚持自己的决策    ○ 放弃这个决定以免受到损失和担心    ○ 没有过以上行为 |
|  |
| **21.**您一般从哪种途径获取投资信息： [单选题] [必答题] |
| ○ 报刊杂志    ○ 电视节目    ○ 网络媒体    ○ 熟人推荐 |
|  |
| **22.**您在网上最常在哪些地方浏览投资信息： [多选题] [必答题] |
| □ 股吧论坛    □ 名人博客    □ 财经网站（如：和讯财经、东方财富、新浪财经等。不包括其下属的股吧论坛）     □ QQ    □ 专业软件（如：同花顺、东方财富通等）     □ 微博    □ 公司官网（如：上市公司网站等） |
|  |
| **23.**这其中您认为对您决策起到作用最大的是： [单选题] [必答题] |
| ○ 股吧论坛    ○ 名人博客    ○ 财经网站    ○ QQ    ○ 专业软件    ○ 微博    ○ 公司官网 |
|  |
| **24.**您在这些媒体上最关注哪个版块： [单选题] [必答题] |
| ○ 公司财报    ○ 熟人推荐    ○ 股评分析    ○ 网友爆料    ○ 新闻公告    ○ 其他 |
|  |
| **25.**您近三年来的投资是否盈利： [单选题] [必答题] |
| ○ 是    ○ 否 |
|  |
| **26.**您认为您的投资结果是否达到了自己的预期： [单选题] [必答题] |
| ○ 达到了预期    ○ 未达到预期 |
|  |
| | 1. 您认为您所受到的外部媒体信息对您的未达预期的消极影响有多大：   [输入0(0%)到100(100%)的数字] [必答题] | | --- | |
|  |
| | 1. 您认为其中您所选用的外部信息媒体对您达到预期的积极影响有多大：   [输入0(0%)到100(100%)的数字] [必答题] | | --- | |
|  |
| **您已完成本次问卷调查的全部内容！  再次感谢您对我们调查的配合与支持，祝您在以后的投资过程中取得更加辉煌的成功！** |
